# Identifying and targeting determinants of melanoma cellular invasion

**DOI:** 10.18632/oncotarget.9227

**Published:** 2016-05-09

**Authors:** Aparna Jayachandran, Prashanth Prithviraj, Pu-Han Lo, Marzena Walkiewicz, Matthew Anaka, Briannyn L. Woods, BeeShin Tan, Andreas Behren, Jonathan Cebon, Sonja J. McKeown

**Affiliations:** ^1^ Olivia Newton-John Cancer Research Institute, Olivia Newton-John Cancer and Wellness Centre, Heidelberg, Victoria, Australia; ^2^ Ludwig Institute for Cancer Research, Melbourne-Austin Branch, Victoria, Australia; ^3^ Department of Medicine, University of Melbourne, Victoria, Australia; ^4^ School of Cancer Medicine, La Trobe University, Victoria, Australia; ^5^ The University of Queensland School of Medicine and the Gallipoli Medical Research Institute, Greenslopes Private Hospital, Brisbane, Queensland, Australia; ^6^ Department of Anatomy and Neuroscience, University of Melbourne, Victoria, Australia

**Keywords:** embryonic chicken transplantation, melanoma, epithelial-to-mesenchymal transition, invasion

## Abstract

Epithelial-to-mesenchymal transition is a critical process that increases the malignant potential of melanoma by facilitating invasion and dissemination of tumor cells. This study identified genes involved in the regulation of cellular invasion and evaluated whether they can be targeted to inhibit melanoma invasion. We identified *Peroxidasin (PXDN), Netrin 4 (NTN4)* and *GLIS Family Zinc Finger 3 (GLIS3)* genes consistently elevated in invasive mesenchymal-like melanoma cells. These genes and proteins were highly expressed in metastatic melanoma tumors, and gene silencing led to reduced melanoma invasion *in vitro*. Furthermore, migration of *PXDN, NTN4* or *GLIS3* siRNA transfected melanoma cells was inhibited following transplantation into the embryonic chicken neural tube compared to control siRNA transfected melanoma cells. Our study suggests that *PXDN, NTN4* and *GLIS3* play a functional role in promoting melanoma cellular invasion, and therapeutic approaches directed toward inhibiting the action of these proteins may reduce the incidence or progression of metastasis in melanoma patients.

## INTRODUCTION

The vast majority of deaths from melanoma follow from the acquisition of invasive behaviour and the establishment of metastases [1, 2]. While tumor invasion is a critical event in melanoma metastasis, the molecular determinants remain largely unknown. A broader understanding of the mechanism of invasion will generate novel treatment strategies. Cancer cells usually acquire invasive ability through a phenotypic switch called epithelial-to-mesenchymal transition (EMT) [3]. As a consequence of EMT, stationary epithelial cells acquire a mesenchymal phenotype and the ability to migrate and invade during developmental morphogenesis and tumor metastasis [4, 5]. Although melanoma is not an epithelial cancer, recent evidence suggests that melanoma cells exhibit many hallmarks of EMT and conform to the EMT dichotomy of epithelial- and mesenchymal-like phenotypes [6–9]. These two distinct melanoma cell phenotypes differ in their *in vitro* invasive potential and phenotypic switching between these states has been proposed as a mechanism for tumor progression [10].

The EMT program in tumor metastasis includes many morphological and molecular features found in embryonic cells undergoing EMT and invasion [11, 12]. Notably, melanoma cells often show high levels of many molecules reminiscent of the EMT programs undertaken by their embryonic precursor; the neural crest cells (NCCs), a collection of multipotent and migratory cells [13–15]. Studies have also revealed that melanoma cells can revert to a neural crest-like state during metastasis [16, 17]. Consequently, the embryonic chicken transplantation model has emerged as a powerful *in vivo* system for assessing the invasive behaviour and plasticity of melanoma cells [8, 14–16, 18–21]. It involves injecting melanoma cells into a microenvironment that is populated with neural crest cells that undergo an EMT to exit from the neural tube and undergo extensive migration, eventually populating a diversity of areas in the embryo [22, 23]. Melanoma cells transplanted into this model respond to cues within the host embryonic microenvironment, do not form tumors, and subsequently mimic many aspects of neural crest cell motility [17, 19]. The embryonic chicken transplantation model has consequently been utilized to investigate the role of candidate genes in motility and pathfinding *in vivo* by perturbing gene expression with morpholino or siRNA [8, 14, 15, 21].

We propose that genes over-expressed in mesenchymal-like melanoma cell lines that exhibit an invasive phenotype are valid targets for blocking invasion *in vitro* and *in vivo*. The aim of our study was to identify candidate genes modulating melanoma cellular invasion. We also investigated whether the candidate genes can be targeted in order to impede melanoma invasion. We have previously utilized a microarray screen to identify genetic transcripts that were over-expressed in mesenchymal-like as compared to epithelial-like melanoma cells [8]. In this study we investigated three genes consistently upregulated in mesenchymal-like melanoma cells including *Peroxidasin* (*PXDN*), *Netrin 4*(*NTN4*) and *GLIS Family Zinc Finger 3* (*GLIS3*). *PXDN*, the human homologue of the Drosophila gene Peroxidasin is a cell surface peroxidase associated with the extracellular matrix. NTN4 belongs to a family of secreted extracellular proteins. *GLIS3* is a member of the GLI-similar zinc finger protein family and encodes a nuclear protein with five C2H2-type zinc finger domains. These candidate gene expressions were validated in clinical melanoma samples. We applied small interfering RNA (siRNA) approach to examine the silencing effect of candidate genes on melanoma cellular invasion *in vitro*. Subsequently, we utilized the chicken embryo model to evaluate the efficacy of candidate gene silencing on melanoma motility *in vivo*. To our knowledge, an in-depth study of the role of *PXDN*, *NTN4* and *GLIS3* in melanoma invasion has not been performed previously and these genes/proteins may be potential drug targets to block melanoma invasion.

## RESULTS

### Transplantation of melanoma cells into the chicken embryo results in the induction of a motile phenotype

We have previously reported the classification of metastatic human melanoma cell lines into epithelial- and mesenchymal-like based on gene expression profiling and functional assays [8]. To compare the motile behaviour of these human metastatic melanoma cell lines, we utilized the *in vitro* transwell invasion assay and the *in vivo* embryonic chicken transplantation model. We chose to evaluate ten different melanoma cell lines that were derived from resected melanoma metastases from different locations, as depicted in Table 1. We first evaluated the invasive capabilites of these cell lines using an *in vitro* transwell invasion assay with reconstituted Matrigel in Boyden chamber inserts. Mesenchymal-like melanoma cell lines LM-MEL-38, -44, -46, -53, and -77 were highly invasive *in vitro*. In contrast to these cell lines, the epithelial-like melanoma cell lines LM-MEL-28, -34, -42, -62 and -71 lacked invasive ability *in vitro* (data not shown). Invasive abilities of some of these cell lines have been previously reported [8, 21].

**Table 1:** Characteristics of melanoma cell lines

Cell Line	Origin of melanoma cell line	EMT classification	BRAF mutation status
LM-MEL-28	Lymph node metastasis (Groin metastasis)	Epithelial-like	V600E
LM-MEL-34	Lymph node metastasis (Left axillary)	Epithelial-like	WT
LM-MEL-38	Lymph node metastasis (Intra-abdominal)	Epithelial-like	V600E
LM-MEL-42	Splenic metastasis	Epithelial-like	V600K
LM-MEL-44	Rectal metastasis	Mesenchymal-like	WT
LM-MEL-46	Cardiac metastasis	Mesenchymal-like	V600E
LM-MEL-53	Joint metastasis (Shoulder joint and soft tissue)	Mesenchymal-like	WT
LM-MEL-62	Lymph node metastasis (Right axillary)	Epithelial-like	G469E
LM-MEL-71	Brain metastasis	Epithelial-like	V600K
LM-MEL-77	Brain metastasis	Mesenchymal-like	WT

**Figure 1:**
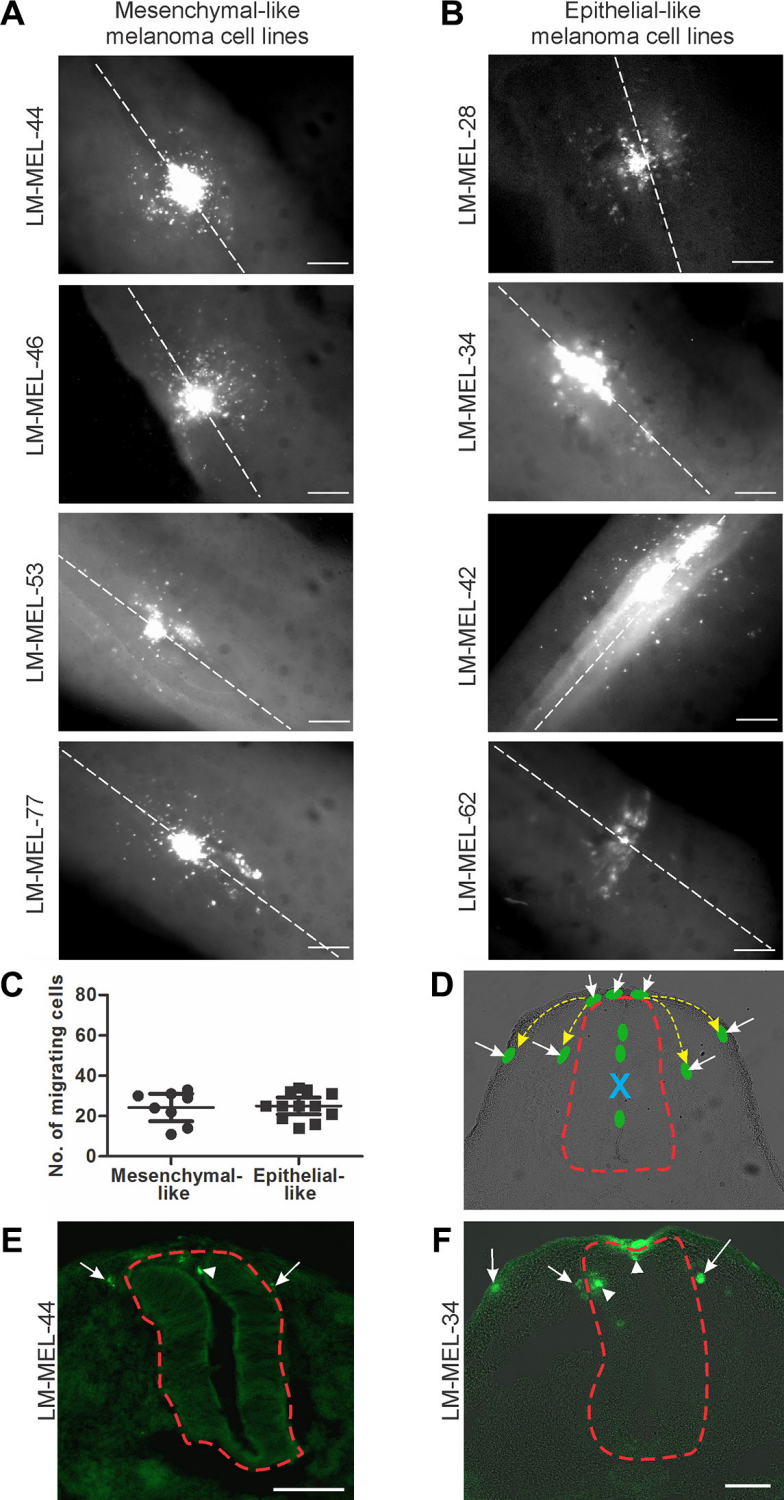
Chick embryo confers invasive properties on poorly invasive melanoma cells Melanoma cells were treated with CM-DiO and cultured as hanging drops to encourage aggregate formation. Similar sized aggregates were introduced into the neural tube of developing chicken and re-incubated within the egg for 2 days. Embryos injected with (**A**) mesenchymal-like melanoma cell lines LM- MEL-44, -46, -53 and -77 and (**B**) epithelial-like melanoma cell lines LM-MEL-28, -34, -42, and -62 were harvested and fluorescence pictures from whole-mounts taken (scale bar = 50 μm). White dotted line indicates the midline of the neural tube. (**C**) From wholemount images, the cells that migrated away from the neural tube were counted. There was no difference between the number of cells migrating from epithelial-like or mesenchymal-like cell lines. (**D**) Representative cross-section of chick embryo with schematic melanoma cells represented by green ovals. Yellow dotted arrows indicate typical migratory pathways of neural crest cells, underneath the ectoderm or by the neural tube. Red dotted line outlines the neural tube. Dorsal is to the top. Site of injection is indicated by blue X and the cells that have moved out of the neural tube are indicated by white arrows. (**E**, **F**) Cross-sections of trunk embryos showing location of melanoma cells (green) from mesenchymal-like cell line LM-MEL-44 (E) and epithelial-like melanoma cell line LM-MEL-34 (F). Arrows indicate motile melanoma cells located outside the neural tube and arrowheads indicate cells remaining inside the neural tube. The neural tube is outlined by a dotted red line. (scale bar = 100 μm).

To evaluate the behaviours of these cell lines in the embryonic chicken transplantation model, we fluorescently-labelled melanoma cells and cultured them as hanging drops overnight to allow the aggregation of cells. Interestingly, mesenchymal-like melanoma cell lines formed dense clusters that were tightly packed together whereas epithelial-like cell lines formed clusters that were loosely packed. These cells were transplanted into the chick neural tube at the trunk level of the developing embryo. The migratory ability of melanoma cells was assessed 2 days post-injection in wholemount embryos and cross-sections (Figure 1). Within the embryonic microenvironment, both invasive mesenchymal-like and non-invasive epithelial-like melanoma cells exit from the site of injection in the neural tube, acquire motile abilities and migrate into surrounding tissues (Figure 1A, 1B). We counted the number of cells that migrated away from the neural tube from both epithelial-like and mesenchymal-like cell lines, and found no difference between these two types of cell lines (Figure 1C). Neural crest cells migrate from the trunk neural tube and follow two main pathways, one ventrally, beside the neural tube, and the other under the ectoderm [20, 24]. Representative cross-section of embryos injected with a mesenchymal-like and an epithelial-like melanoma cell line show no differences in the routes followed by the melanoma cells within the embryos, and the routes are largely the same as those followed by neural crest cells; cells were observed beside the neural tube and underneath the ectoderm (Figure 1D–1F). The timing and the trajectories of melanoma cells do not show apparent differences between epithelial-like and mesenchymal-like melanoma cells. This behaviour is independent of the mutation status of the melanoma cell lines, since the same observation was made using cell lines that were wild-type and cell lines carrying the V600E BRAF mutation (Table 1). Furthermore, no differences in invasive capacities was noted in melanoma cells derived from different metastases, namely, brain, spleen, lymph nodes, rectum, joint, cardiac and skin metastases (Table 1). Taken together, these observations show that melanoma cells can be rewired to acquire a motile phenotype within the chicken embryonic neural microenvironment regardless of their *in vitro* or *in vivo* characteristics.

### siRNA-mediated targeting of transcription factor *Snail* inhibited melanoma invasion

We propose that targeted silencing of genes associated with the invasive melanoma phenotype can be a strategy for blocking melanoma metastasis. As a proof of principle, we first inhibited the expression of the zinc finger transcription factors *Snail* (*SNAI1*) and *Slug* (*SNAI2*). These have been established as crucial regulators of EMT during embryonic development, organ fibrosis and cancer progression, as they are potent repressors of E-cadherin expression and enhancers of migration and invasion [5, 25]. In melanoma cells, both these transcription factors have been implicated in inducing an EMT-like process that would be expected to enhance invasion, tumor progression and metastasis *in vivo* [26–30].

**Figure 2:**
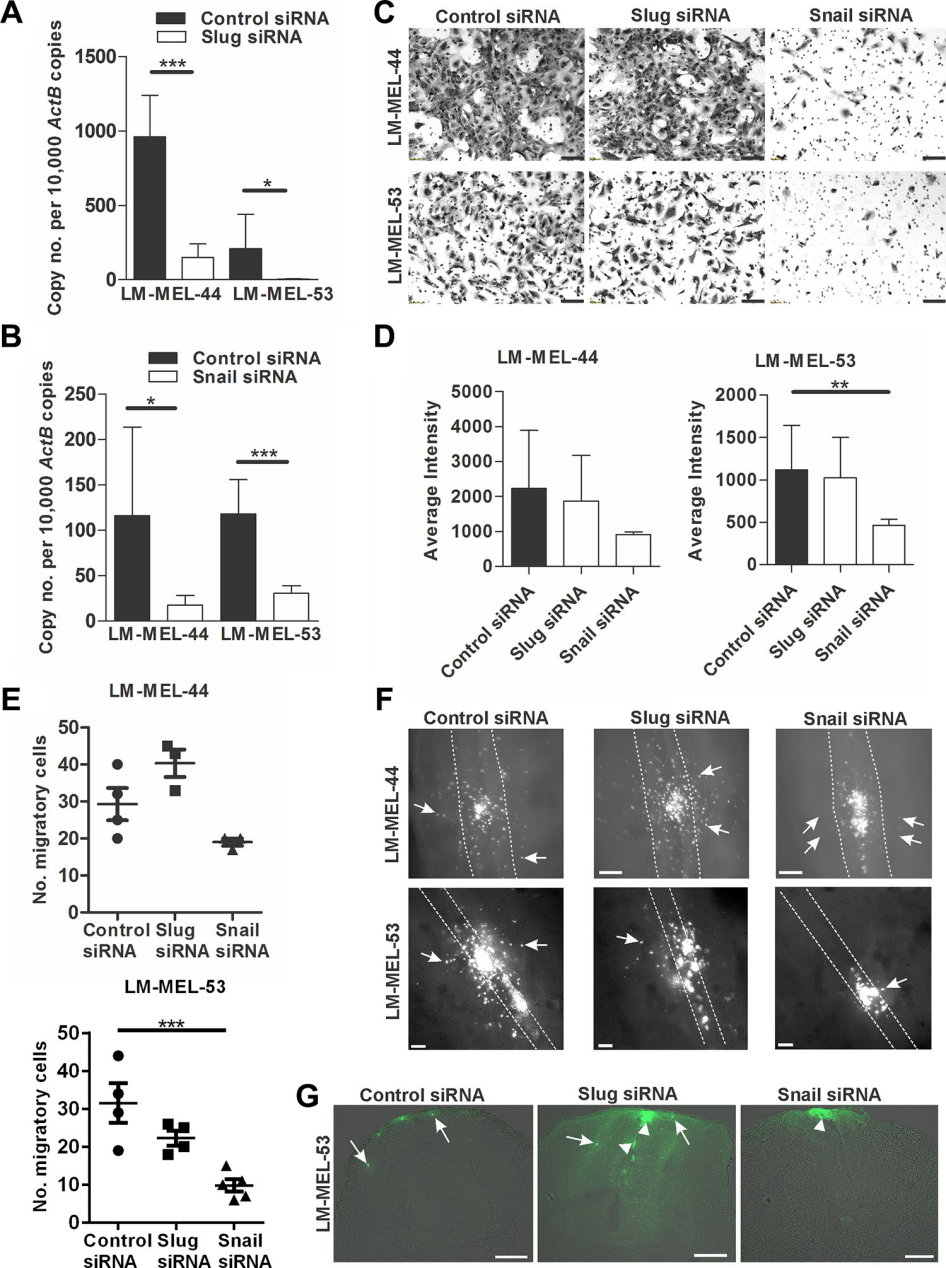
Depletion of snail inhibits melanoma invasion *in vitro* and *in vivo* Melanoma cells were plated out and transfected with either 10 nM control siRNA, *Snail* or *Slug* specific siRNA. After 72 h RNA was extracted and *Slug* (**A**) and *Snail* (**B**) qRT-PCR was performed on melanoma lines LM-MEL-44 and LM-MEL-53. Difference in gene expression was analysed using Student's *t*-test (*p < .05, ***p < .0005). Error bars indicate SEM of three experiments in triplicate. (**C**) Melanoma cells from LM-MEL-44 and -53 were transfected as described and seeded in a Matrigel coated transwell for 24 h. Cells that invaded the transwell membrane were stained with 1% crystal violet and representative images of the membrane were captured (scale bar = 100 μm). (**D**) Quantitative analysis of the number of invasive cells on the transwell membrane was determined by measuring the average intensities of invasive cells calculated in K counts mm^2^ using Odyssey Software. Error bars indicate SEM of three independent experiments in triplicate. Data was analysed using ANOVA with post-hoc Tukey test (**p < .005). (**E–G**) Melanoma cells were labelled with CM-DiO, transfected with the indicated siRNAs, cultured as hanging drops and introduced into the trunk neural tube of chicken embryos. After 2 days embryos were harvested and fluorescence pictures taken from whole-mounts. (E) The number of cells that migrated out of the neural tube was counted. Bars indicate mean +/− SEM. This data was combined with data using the same cell lines from Figure 7 and analysed using ANOVA with post-hoc Tukey test. Significantly fewer *Snail* siRNA treated cells migrated from the neural tube compared to control siRNA treated cells using LM-MEL-53 (*p < .05). (F) Whole-mount dorsal images of representative embryos (scale bar = 100 μm). White dotted lines show the outline of the neural tube and the white arrows indicate fluorescent melanoma cells that migrated out of the neural tube and into the surrounding tissue. (G) Images from cross-section of embryos show motility of melanoma cells. Arrows indicate the motile melanoma cells, arrowheads point to melanoma cells that remain inside the neural tube (scale bar = 100 μm).

RNA interference *in vitro* was used to evaluate the role of *Snail* and *Slug* in melanoma invasion. siRNA treatment silenced *Slug* and *Snail* expression in two invasive mesenchymal-like cell lines, LM-MEL-44 and -53 (Figure 2A–2B). We tested the impact of siRNA treatment on invasion using an *in vitro* transwell Matrigel assay. Fewer cells invaded through the Matrigel onto the transwell membrane in LM-MEL-53 transfected with *Snail* siRNA, as compared with the control or *Slug* siRNA treated cells, visualised by images of the invasive cells stained with 1% crystal violet and quantitative analysis of the intensity of cell staining (Figure 2C–2D). Next, we assessed *Snail* and *Slug* function in the embryonic chicken model. Melanoma cells were transfected with siRNA targeting *Snail* or *Slug*, cultured as a hanging drop for 24 hours and introduced into the trunk neural tube of a developing chick embryo. *Snail* siRNA treated melanoma cells demonstrated a significant reduction in emigration from the neural tube *in vivo* into the surrounding tissue (Figure 2E–2G). In contrast, control and *Slug* siRNA treated melanoma cells migrated into the surrounding mesenchymal host tissue (Figure 2E–2G). The number of melanoma cells emigrating out of the neural tube was counted, and silencing of *Snail* resulted in the least number of cells exiting the neural tube when compared to control or *Slug* siRNA treated melanoma cells (Figure 2E). These proof-of-concept experiments demonstrated that siRNA-mediated silencing of *Snail*, but not *Slug* in invasive mesenchymal-like melanoma cell lines lead to a marked decrease in motility *in vitro* and *in vivo*.

### Identification of candidate genes for targeting invasion in melanoma cells

We identified potential candidate genes whose function are related to melanoma cell invasion from a previously generated whole genome microarray expression profiling study that compared gene expression of epithelial- and mesenchymal-like melanoma cell lines [8]. Among the 221 differentially expressed genes in mesenchymal-like melanoma cells, 65 genes were selected based on their putative role in regulation of cell plasticity and invasion in development or disease, as determined by literature review. These EMT and invasion related genes included extracellular matrix (ECM) components, membrane bound receptors, cytosolic proteins and transcription factors. To validate the expression of the selected candidate genes in melanoma, we compared their expression by qRT-PCR across a subset of nine mesenchymal- and nine epithelial-like melanoma cell lines (data not shown).

In the present study, we selected genes which were highly expressed in at least 6 of 9 mesenchymal-like melanoma cell lines and showed no or little expression in epithelial-like melanoma cell lines. Numerous candidate genes such as *ECOP1* and *PMEPA1* were eliminated as they were also expressed abundantly in some epithelial-like cell lines (Supplementary Figure S1). Three candidate genes emerged that exhibited varying degrees of higher expression in mesenchymal-like melanoma cell lines. These genes are *Peroxidasin* (*PXDN*), *Netrin 4* (*NTN4*) and *GLIS Family Zinc Finger 3* (*GLIS3*). The expression of *PXDN*, *NTN4* and *GLIS3* are all associated with motility, however these genes have not been previously described as modulators of EMT and invasion in melanoma and have not been evaluated for their potential for contributing to invasion [31–33].

### siRNA-mediated *PXDN*, *NTN4* and *GLIS3* knockdown impaired invasion of melanoma cells *in vitro*

**Figure 3:**
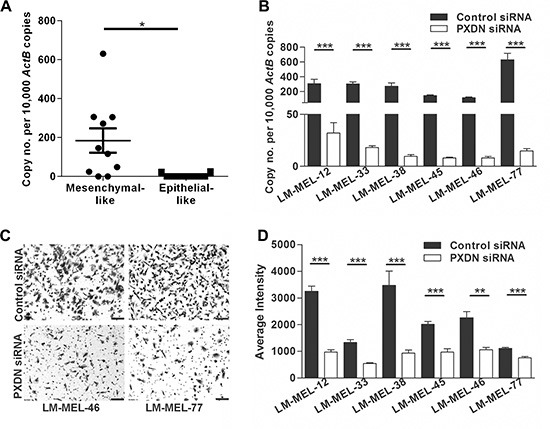
Silencing *PXDN* blocks *in vitro* invasion of melanoma cells (**A**) Using qRT-PCR *PXDN* expression in ten mesenchymal- and epithelial-like melanoma cell lines was analyzed. Bars indicate mean +/− SEM (*t*-test *p < .05). (**B**) Six melanoma cell lines were transfected with either 10 nM control siRNA or *PXDN* specific siRNA and knockdown was evaluated by qRT-PCR after 72 h (*t*-test, ***p < .0005). (**C–D**) The *in vitro* invasive ability of these cells lines was tested using a Matrigel assay. (**C**) Representative images of invasive cells were taken (scale bar = 100 μm) and (D) average intensities of invasive cells after crystal violet staining was calculated in K counts mm^2^ using Odyssey Software. Bars indicate mean +/− SEM of three independent experiments in triplicate. Data was combined with data from same cell lines in Figure 4 and Figure 5 and analysed using ANOVA, with post-hoc Tukey test to identify treatments significantly different from control (**p < .005, ***p < .0005).

High expression of *PXDN* was found in most mesenchymal-like melanoma cell lines compared with epithelial-like melanoma cell lines (Figure 3A). We observed diminished *PXDN* mRNA 72 hours after transfection of *PXDN* specific siRNA but not with a control siRNA in LM-MEL-12, -33, -38 -45, -46, and -77 (Figure 3B). Immunofluorescent staining showed reduction in PXDN expression 72 hours after transfection of PXDN specific siRNA but not with a control siRNA (Supplementary Figure S2A). We next evaluated effects of PXDN knockdown on cellular invasion. Invasion is the early crucial step of the metastatic cascade and model systems have been developed to recapitulate melanoma cellular invasion [34, 35]. Transwell invasion assays using the reconstituted Matrigel in Boyden chamber inserts have been utilized to study melanoma cell invasion *in vitro* [8, 9, 27]. Transfection with a control siRNA did not alter the invasive ability of mesenchymal-like melanoma cell lines, while PXDN silencing in these cell lines resulted in a significant reduction of invasion (Figure 3C–3D).

**Figure 4:**
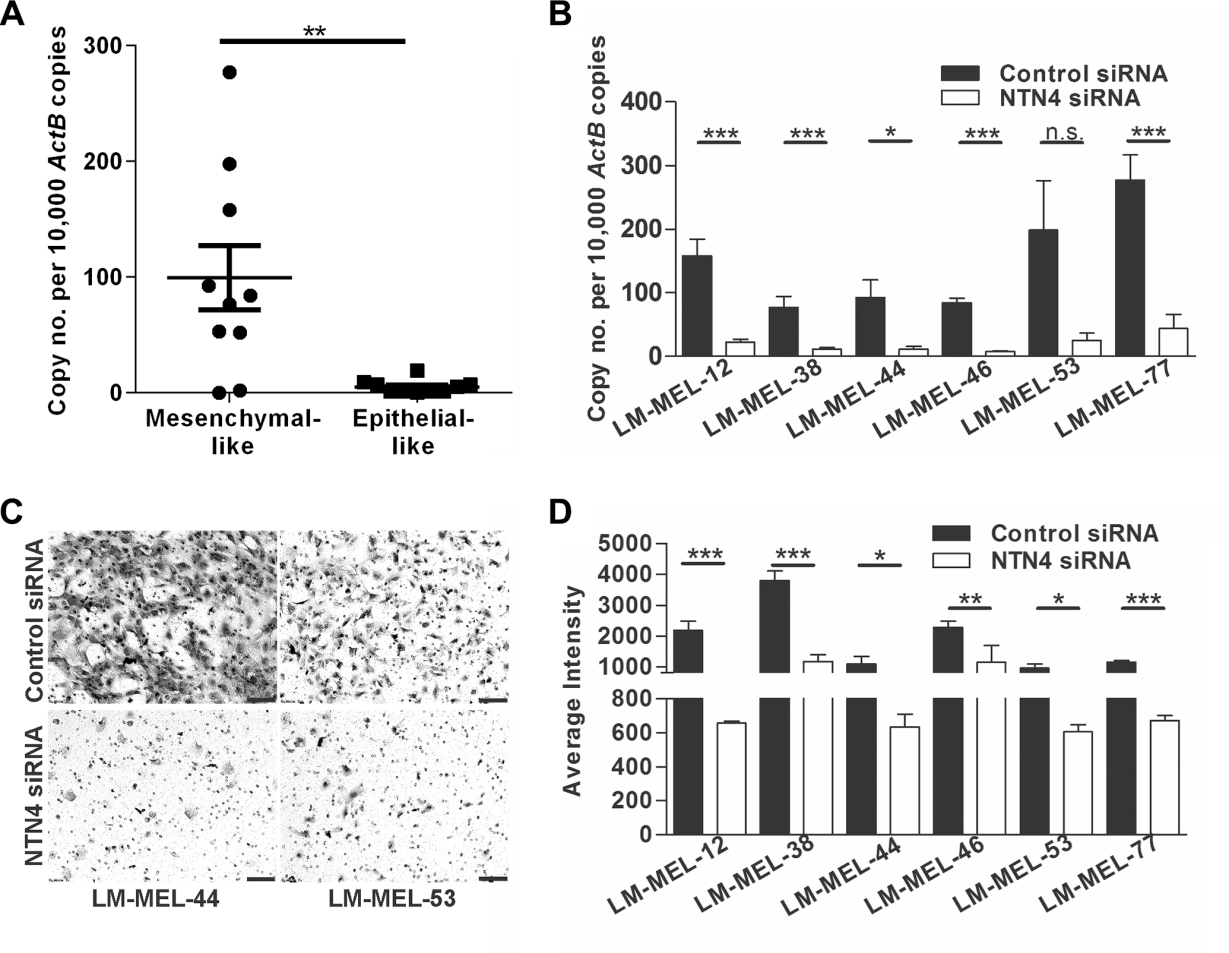
Targeting *NTN4* results in loss of invasive potential of melanoma cells *in vitro* (**A**) *NTN4* expression in ten mesenchymal- and epithelial-like melanoma cell lines was tested by qRT-PCR. Bars indicate mean +/− SEM (*t*-test, **p < .005). (**B**) Melanoma cell lines were transfected with either 10 nM control siRNA or *NTN4* specific siRNA and *NTN4* qRT-PCR was performed after 72 h (*t*-test, *p < .05, ***p < .0005, n.s = not significant). (**C–D**) The *in vitro* invasive ability of these cells lines was tested using a Matrigel assay. (C) Representative images of invasive cells were taken (scale bar = 100 μm) and (D) average intensities of invasive cells calculated in K counts mm^2^ using Odyssey Software. Bars indicate mean +/− SEM of three independent experiments in triplicate. Data was combined with data from same cell lines in Figure 3 and Figure 5 and analysed using ANOVA, with post-hoc Tukey test to identify treatments significantly different from control (*p < .05, **p < .005, ***p < .0005).

*NTN4* expression was significantly higher in most mesenchymal-like melanoma cells compared with epithelial-like melanoma cells (Figure 4A). Two different siRNAs were used in order to control nonspecific effects and tested in six different melanoma cell lines. *NTN4* was efficiently inhibited at 72 hours after siRNA transfection in all cell lines tested, namely LM-MEL-12, -38, -44, -46, -53, and -77 (Figure 4B). Reduction in NTN4 expression was also determined by immunofluorescent staining (Supplementary Figure S2B). Silencing NTN4 reduced the invasive ability of mesenchymal-like melanoma cells compared with control siRNA treated cells *in vitro* (Figure 4C–4D).

**Figure 5:**
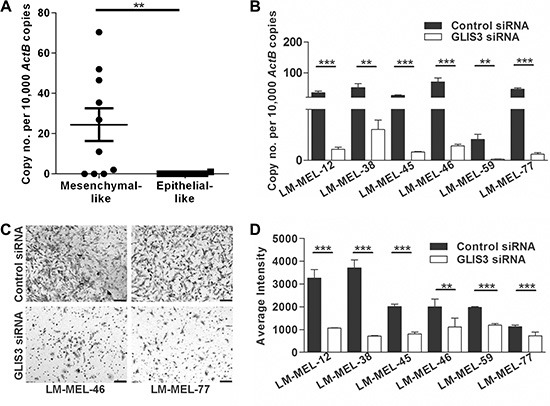
Silencing *GLIS3* results in the abrogation of melanoma invasion *in vitro* (**A**) *GLIS3* expression was evaluated in ten mesenchymal- and epithelial-like melanoma cells by qRT-PCR. Bars indicate mean +/− SEM (*t*-test, **p < .05). (**B**) 72 h after transfection of six melanoma cell lines with either 10 nM control siRNA or *GLIS3* specific siRNA *GLIS3* qRT-PCR was performed (*t*-test, **p < .005, ***p < .0005). (**C–D**) The *in vitro* invasive ability of these cells lines was tested using a Matrigel assay. (C) Representative images of invasive cells were taken (scale bar = 100 μm). (D) Average intensities of invasive cells were calculated in K counts mm^2^ using Odyssey Software. Bars indicate mean +/− SEM of three independent experiments in triplicate. Data was combined with data from same cell lines in Figure 3 and Figure 4 and analysed using ANOVA, with post-hoc Tukey test to identify treatments significantly different from control (**p < .005, ***p < .0005).

*GLIS3* was also found to be upregulated in the majority of mesenchymal-like melanoma cells compared with epithelial-like melanoma cells that expressed very little or no *GLIS3* (Figure 5A). We next decreased *GLIS3* expression using two different siRNAs. *GLIS3* expression levels in six mesenchymal-like melanoma cells LM-MEL-12, -38, -46, -45, -59 and -77 was effectively down-regulated (Figure 5B). We further validated reduction of GLIS3 protein expression by immunofluorescent staining (Supplementary Figure S2C). We then analyzed the invasive behaviour of melanoma cells after suppression of GLIS3. We determined that the reduction of GLIS3 expression suppressed melanoma cell invasion *in vitro* (Figure 5C–5D). All of the examined genes may therefore play an important role in promoting or establishing melanoma cell invasion.

### Metastatic melanomas express large amounts of PXDN, NTN4 and GLIS3

**Figure 6:**
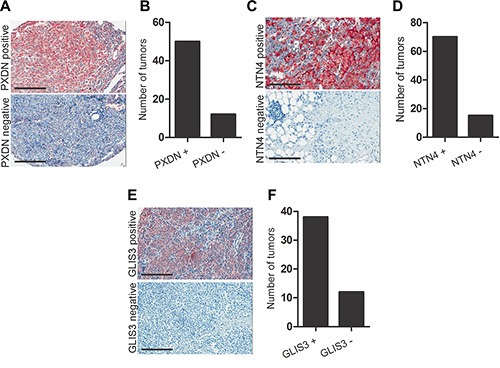
PXDN, NTN4 and GLIS3 immunostaining in melanoma tumor tissue Localization of (**A**) PXDN, (**C**) NTN4 and (**E**) GLIS3 in metastatic melanoma tumor biopsies (scale bar = 50 μm). Graph shows number of tumors scored for (**B**) PXDN, (**D**) NTN4 and (**F**) GLIS3 expression.

Given that *PXDN*, *NTN4* and *GLIS3* can regulate invasion of melanoma cells *in vitro*, we evaluated the *in vivo* relevance of our data in melanoma tumors. We examined the expression of these genes in a panel of 46 metastatic melanoma patient tumours and detected expression of all three genes in the majority of the samples tested (Supplementary Figure S3A–S3C). We have assessed correlations between the three genes in patient samples and found significant but weak correlation between *GLIS3* and *PXDN* expression, and between *GLIS3* and *NTN4*. No correlation in expression was noted between *NTN4* and *PXDN* (Supplementary Figure S4A– S4C). We then analysed the protein expression patterns of PXDN, NTN4 and GLIS3 in melanoma tumors by immunohistochemical staining of tissue microarrays (TMA) comprising tumors from patients with stage III and IV metastatic melanoma. PXDN expression was detected in 81% (50/62) of metastatic melanoma patient tumors (Figure 6A–6B). The subcellular location was identified as predominantly membranous. NTN4 expression was detected as both cytoplasmic and membranous in 85% (70/85) of metastatic melanoma tumors (Figure 6C–6D). GLIS3 was detected as cytoplasmic and nuclear in 76% (38/50) of metastatic melanoma tumors (Figure 6E–6F). Placenta was used as positive control tissue for PXDN, testis was used as positive control for NTN4 and prostate tissue was used as a positive control for GLIS3 staining (Supplementary Figure S5).

### Expression of PXDN, NTN4 and GLIS3 is associated with markers of EMT

Cadherin switching is a hallmark of EMT, leading to the down-regulation and replacement of the cell surface adhesion molecule E-cadherin by N-cadherin, enabling motility [3, 8]. Since PXDN, NTN4 and GLIS3 are involved in motility and associated with mesenchymal gene signature in melanoma cell lines, we investigated the association of these candidate proteins with markers of EMT in clinical samples.

A cutaneous melanoma dataset consisting of 421 melanoma patients available from The Cancer Genome Atlas (TCGA) (http://www.cbioportal.org) was analysed for association of PXDN, NTN4 and GLIS3 with EMT markers. Large-scale proteomics data from the reverse phase protein array (RPPA) platform are available in the portal for TCGA melanoma studies. For each available protein, the Portal performs a two-sided, two-sample Student's *t* test to identify differences in protein abundance between tumor samples that have at least one event (alteration) in one of the query genes, and those that do not [36, 37]. Analysis revealed that a subset of patients with altered (upregulated) PXDN showed significantly low E-cadherin (*p*-value: 8.91e-6) and high N-cadherin (*p*-value: .01) protein expression compared with patients with unaltered PXDN expression (Supplementary Figure S6). Patients with altered (upregulated) NTN4 showed no difference in E-cadherin (*p*-value: .5) and N-cadherin (*p*-value: .1) protein expression (Supplementary Figure S7). In patients with altered (upregulated) GLIS3 significantly low E-cadherin (*p*-value: 2.53e-6) and high N-cadherin (*p*-value: .0046) protein expression was detected (Supplementary Figure S8). The significant correlation between PXDN and GLIS3 expression with EMT markers that was observed in our melanoma cell line studies is therefore also confirmed in a large clinical tumor dataset. In TCGA melanoma dataset, mutual exclusivity data from melanoma patients revealed that both PXDN and GLIS3 are co-expressed (Odds ratio = 2.23, *p* = .010, Fisher's exact test).

### Migratory behaviour of melanoma cells in the chicken transplantation model is reduced following knockdown of PXDN, NTN4 or GLIS3

**Figure 7:**
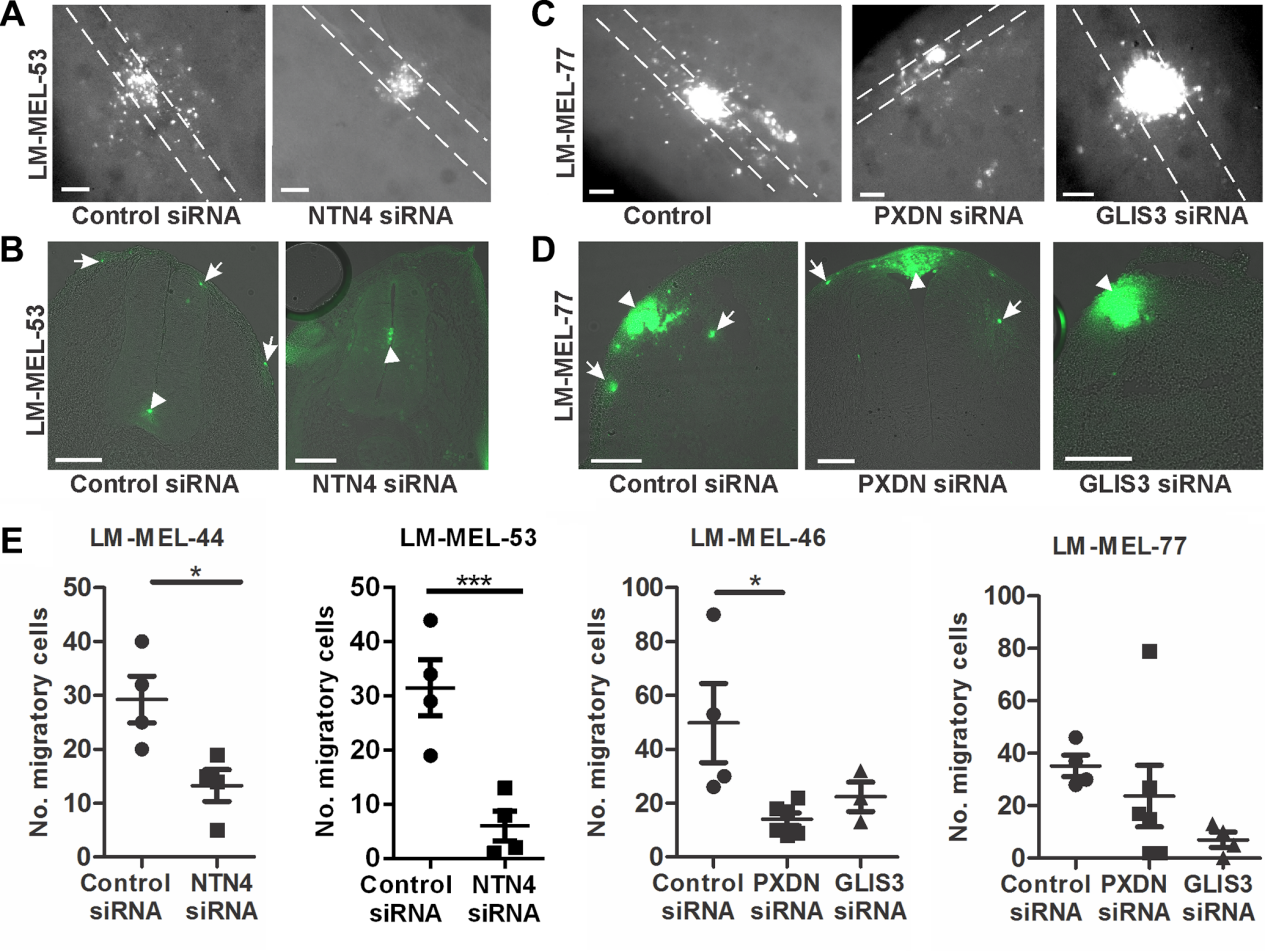
Depletion of PXDN, NTN4 and GLIS3 inhibits melanoma invasion in the chicken transplantation model Melanoma cells were treated with CM-Dio and transfected with either 10 nM control siRNA, *PXDN*, *NTN4* or *GLIS3* specific siRNA. Cells were cultured as hanging drops and introduced into the trunk neural tube of 2 day chicken embryos (Hamburger-Hamilton stages 11 to 14). (**A**) Whole mount images of embryos transplanted with control or *NTN4* siRNA treated LM-MEL-53. White dotted lines show the outline of the neural tube. (**B**) Cross section of embryos showing motility of melanoma cells after control or *NTN4* siRNA treatment. Arrows indicate motile melanoma cells, arrowheads show melanoma cells that remain in the neural tube. (**C**) Whole mount images of embryos transplanted with control or *GLIS3* or *PXDN* siRNA in LM-MEL-77. White dotted lines show the outline of the neural tube. (**D**) Cross-section of embryos showing motility of melanoma cells after control or *PXDN* or *GLIS3* siRNA treatment. Arrows indicate the motile melanoma cells, and arrowheads show melanoma cells that remain at the site of transplantation. (**E**) The number of cells outside the neural tube from each embryo was counted and plotted for LM-MEL-44 and -53 after *NTN4* or control siRNA treatment. The same controls were used in the *Slug* and *Snail* siRNA treatments (Figure 2E) and these data were analysed together using ANOVA with post-hoc Tukey test (*p <.05). The number of cells outside the neural tube from each embryo was counted and plotted for LM-MEL-77 and -46 after *GLIS3*, *PXDN* or control siRNA treatments. Lines indicate mean +/− SEM, (Anova with post-hoc Tukey test, *p <.05). Scale bars = 100 μm.

The preceding experiments indicated that PXDN, NTN4 and GLIS3 are highly expressed in metastatic melanoma and play a role in promoting melanoma cell invasion *in vitro*. To further assess this at a functional level we used the chicken transplantation model to evaluate the roles of these candidate genes. Melanoma cells were transfected with siRNA targeting *PXDN*, *NTN4* or *GLIS3*, cultured as a hanging drop for 24 hours and introduced into the trunk neural tube of a developing chick embryo. *NTN4* siRNA treated melanoma cells from LM-MEL-44 and -53 predominantly remain at the site of injection and demonstrated a significant reduction in emigration from the neural tube *in vivo* into the surrounding tissue in the embryo in whole mount and cross-sections (Figure 7A–7B). In contrast numerous control siRNA treated cells migrate out of the neural tube (Figure 7A–7B). Whole mount and cross-sections of embryos shows that fewer *PXDN* and *GLIS3* siRNA treated melanoma cells migrated out of the neural tube compared to control siRNA treated cells in LM-MEL-77 (Figure 7C–7D) and -46 (not shown). Counts of the number of cells outside the neural tube showed that significantly fewer NTN4 siRNA treated cells migrated compared to controls from cell lines LM-MEL-44 and -53, while significantly fewer PXDN siRNA treated cells migrated in cell line LM-MEL-46 and there was a trend towards fewer migratory GLIS3 siRNA treated cells in LM-MEL-77 compared to control siRNA treated cells. These observations strongly indicated that *PXDN*, *NTN4* and *GLIS3* expression is associated with invasive ability in melanoma cells.

## DISCUSSION

Despite extensive studies on melanoma, the molecular mechanisms of melanoma tumor invasion are still not fully understood. Through a knockdown approach and *in vivo* motility model, we demonstrate 3 novel molecular players *PXDN*, *NTN4* and *GLIS3* as critical factors of melanoma invasion. Our results show that PXDN, NTN4 and GLIS3are overexpressed in metastatic melanoma clinical specimens. These genes promote motility, a crucial cellular feature associated with metastatic disease, thus implicating these gene products as potential drug targets to block melanoma cell motility.

Metastasis is a complex multi-step event and invasion of a tumor through basement membrane into surrounding tissue is the first crucial step of the metastasis process [4, 38]. We and others have previously shown that in melanoma, invasion and proliferation are uncoupled, such that highly proliferative melanoma cells are less likely to be invasive, and vice versa [8, 10, 39], but the properties are not mutually exclusive, and invading cells undergo proliferation [40]. The transition between each state is referred to as phenotype switching, which is similar to the EMT process. Many studies have shown that melanoma cells with mesenchymal traits such as a heightened ability to invade (as detected by *in vitro* Matrigel coated Boyden based invasion chamber assays) possessed increased tumor metastasis *in vivo* in mouse models of melanoma [27, 30, 34, 41]. Furthermore, microarray profiling of the melanoma tumor derived from patients reveals characteristic invasive and mesenchymal expression signatures associated with metastatic outcome [42]. Such observations are consistent with a crucial role for invasion in melanoma metastasis. In this study we have identified 3 candidate genes that functionally correlated with heightened invasiveness in mesenchymal-like melanoma cells. Knocking down expression of each of these genes reduced cell motility *in vitro* and *in vivo*; therefore further investigation of the role of these genes in cell invasion and other aggressive melanoma traits such as drug resistance, adhesion and proliferation is warranted.

Studies using gene expression arrays have revealed coordinated upregulation of numerous genes involved in cell motility in mesenchymal-like melanoma cell lines. This coordinated upregulation of genes probably reflects the activation of EMT transcriptional programs that promote cell motility by either environmental cues such as matrix components present in mesenchymal-like melanoma cells or by changes in some master regulatory genes [8, 42]. The invasive behaviour of melanoma cells has been mainly attributed to their origin from the embryonic neural crest cells [17]. Hence, the embryonic microenvironment populated with neural crest cells is considered to be an ideal and clinically relevant micro-compartment for *in vivo* studies of melanoma cells [16, 43–45].

The chicken transplantation model has generated new ways to understand the basic biology of melanoma motility and to test strategies that aim to block the spread of melanoma [8, 14, 15, 20, 21]. Tumor cells are either intrinsically able to migrate (mesenchymal cells) or acquire this capability during tumor progression as part of promigratory programs such as EMT, often in response to microenvironmental cues [46]. When transplanted into the chicken embryonic neural crest microenvironment, melanoma cells respond to cues from surrounding host tissues and follow migratory routes travelled by NCCs [16]. We demonstrate that 10 different melanoma cell lines regardless of their site of metastasis, BRAF mutation status or invasive behaviour *in vitro*, gain motility when transplanted into the trunk chicken microenvironment, reflecting the underlying plasticity of these cell types.

Interestingly, we observed that mesenchymal-like melanoma cell lines formed smoother and more tightly packed clusters when cultured as a hanging drop compared to epithelial-like cell lines. We have previously shown that culturing both epithelial- and mesenchymal-like melanoma cell lines as hanging drops does not promote an EMT [20]. However, this difference in appearance between the cell lines in hanging drops was not associated with differences in migration, as melanoma cells from both epithelial and mesenchymal-like lines migrated following transplantation into chick trunk neural tube. It is likely that the melanoma cells exhibit motility *in vivo* by undergoing an EMT process in response to the chick microenvironmental cues. This is in accordance with a recent study demonstrating that 13 known neural crest genes associated with EMT and cell migration were induced in melanoma cells following exposure to the neural crest microenvironment [15, 47]. Assessing changes in EMT traits after harvesting melanoma cells exposed to chick microenvironment warrants in-depth investigation.

Melanomas modify their microenvironment through secretion of specific extracellular matrix proteins and extracellular matrix modifying proteases to promote tumor growth and metastasis [48]. Alterations in extracellular matrix remodelling have been shown to be an important event during EMT [46]. Two of the candidate genes identified in this study are extracellular matrix proteins, namely PXDN and NTN4. PXDN is a unique member of the heme-containing peroxidases enzyme family that was initially identified to play a role in embryonic development in *Drosophila melanogaster* [49]. PXDN is involved in the formation and stabilization of the ECM in a way that does not seem to be mediated by its peroxidase activity [50]. In addition to melanoma, PXDN was also detected in tumors such as breast cancer, colon cancer, glioblastoma and ovarian cancer [51, 52]. A gene-set enrichment analysis (GSEA) of our previous study of mesenchymal melanoma cell lines [8] revealed association of PXDN with breast cancer [53] and colon cancer [54]. PXDN, also known as Melanoma-associated gene-50 (*MG50*), encodes a new melanoma antigen containing at least six naturally expressed melanoma HLA class 1-restricted epitopes that are recognized by human cytolytic T lymphocytes, further indicating its potential clinical applications [51]. Although PXDN was expressed in melanoma samples its physiological role remains largely unexplored. Silencing PXDN in mesenchymal melanoma cells blocked cellular invasion and this observation is consistent with a previous study showing that loss of PXDN in BeWo choriocarcinoma cells blocked cellular invasion and adhesion [32]. Transforming growth factor – β1 (TGF-β1) treatment induced the production of PXDN by differentiating myofibroblasts undergoing an EMT during tissue fibrosis [50]. TGF-β1 induced EMT in melanoma by modulating ECM molecules and it is likely that PXDN represents an ECM molecule in melanoma mediating an EMT in a TGF-β1-dependent manner [8, 21]. Since PXDN is a surface protein accessible to extracellular pharmaceutical compounds, it may prove to be a novel target for abrogating tumor invasion in melanoma.

NTN4 is a member of the netrin family that are secreted chemotropic guidance cues with roles in embryogenesis and tumor development [55, 56]. NTN4 is emerging as an important regulator of epithelial and endothelial migration, adhesion, proliferation and apoptosis [57, 58]. In cancers, the expression and functional role of NTN4 has been controversial. Recent studies have suggested a role for NTN4 in regulating metastasis, angiogenesis and tumor growth [31, 58]. However, other studies examining expression of NTN4 in clinical samples have reported that it is markedly down-regulated in prostate, breast and cervical cancers [59–61]. The variable expression patterns of NTN4 in different cancers could depend on many factors including the receptors available on the responsive cells, multiple signaling pathways activated and the concentration of protein [56, 58]. NTN4 functions as an ECM molecule, is a component of basement membranes and regulates cell interaction with the ECM [62]. NTN4 has been implicated in activating multiple oncogenic pathways in gastric cancer, namely Jak/Stat, PI3K/Akt and ERK/MAPK [63], and it interacts with integrin β4 to promote glioblastoma cell proliferation via the AKT-mTor pathway [56]. Our previous GSEA of melanoma cell lines revealed that NTN4 was down-regulated in osteosarcoma cells following knockdown of HDAC2 [64], suggesting that it is epigenetically regulated in cancer cells. Our data suggest a role for NTN4 in regulating melanoma invasion, and NTN4 being an ECM protein, peptides or antibodies that inhibit or limit its action might be among the strategies to control or limit melanoma metastasis.

Gli-similar (GLIS) 1–3 proteins constitute a subfamily of Krüppel-like zinc-finger transcriptional regulators that play a critical role in embryonic development and in several pathologies including kidney fibrosis [65]. During embryonic development GLIS3 regulates neurulation, is prominently expressed in the dorsal neural tube and is implicated in cellular motility [33]. GLIS3 has been reported to be highly expressed in ependymomas and glioblastomas, suggesting a role for GLIS3 in tumorigenesis [66, 67]. GLIS3 interacts with the transcriptional coactivator with PDZ-binding motif (TAZ) which has been implicated in EMT [68, 69]. GLIS3 shares a great degree of homology with members of the Gli family and provides a mechanism for crosstalk between Gli and Glis signaling pathways. Recently, GLI2, a TGF-β target has been implicated in increased melanoma invasion and metastatic capacity [41]. Our previous GSEA of melanoma cell lines revealed association of GLIS3 with breast cancer [53] (the same gene set that showed upregulation of PXDN), supporting its role in multiple tumour types. Elucidation of GLIS3 and its interaction partners in melanoma may provide new opportunities for the development of therapeutic strategies in the treatment of this disease.

Our study suggests that *Snail* (*SNAI1*) rather than *Slug* (*SNAI2*) plays a role during later stages of melanoma invasion and metastasis. This is consistent with reports demonstrating a role for *Slug* early during melanoma development, rather than during later stages of metastasis [9, 26, 30, 39]. This is in contrast to other studies implicating Slug as pro-invasive gene responsible for the metastatic behaviour of melanomas [28, 29, 70]. Increase in the mesenchymal phenotype and metastatic ability of melanomas with Snail overexpression has been demonstrated [71, 72]. Both Snail and Slug are crucial for the neural crest development and functional redundancies between the family members have been well documented [22, 73, 74].

Although this study shows an association between PXDN, NTN4, GLIS3 and SNAI1 and melanoma invasion, their roles in melanoma are most likely multifaceted. We observed some correlation in gene expression in patient samples between *GLIS3* and *PXDN*, and *GLIS3* and *NTN4*. How these proteins are regulated and how they act in metastasis is not yet clear. Thus further understanding of the molecular crosstalks between these proteins and EMT-related signaling pathways may also provide effective approaches to targeting a broader signaling pathway underlying melanoma plasticity.

## MATERIALS AND METHODS

### Cell culture and melanoma patient samples

Melanoma cell lines were established from resected melanoma metastases by mechanical dissociation of tissue with subsequent overnight digestion in media containing collagenase IV at 37°C. Established cell lines were Mycoplasma-tested using the MycoAlert test (Lonza Rockland, Inc., USA). All tissue donors provided written informed consent for tissue collection and research, which was covered by protocols approved by the Austin Health Human Research Ethics Committee, Melbourne, Australia (approval number H2012/04446). All cell lines were matched with their donors by HLA-typing and STR-profiling. Cells were cultured in RPMI1640 supplemented with 10% fetal calf serum (FCS) as described previously [75].

### qRT-PCR

RNA for qPCR was extracted using the RNEasy kit (Qiagen, Germany) or the Acturus^®^ RNA Picopure^®^ kit (Life Technologies, USA). Reverse transcription was carried out using the High Capacity cDNA RT kit (Applied Biosystems, Life Technologies, USA). Following reverse transcription, qRT-PCR was performed using SYBR Green (Qiagen, Germany). Beta-Actin (*ActB*) was used as internal control. Following primers were used: *ActB* (forward) 5′-ctg gaa cgg tga agg tga ca-3′ and (reverse) 5′-cgg cca cat tgt gaa ctt tg-3′, *SNAI1* (forward) 5′-gct gca gga ctc taa tcc aga-3′ and (reverse) 5′-atc tcc gga ggt ggg atg-3′, *SNAI2* (forward) 5′-tgg ttg ctt caa gga cac at-3′ and (reverse) 5′-gtt gca gtg agg gca aga a- 3′, *PXDN* (forward) 5′-tgc aca ata aga gcg aac ca-3′ and (reverse) 5′-tcc tta cat tcg cat tta cct g-3′, *NTN4* (forward) 5′-tgc aca ata aga gcg aac ca-3′ and (reverse) 5′-tcc tta cat tcg cat tta cct g-3′ and *GLIS3* (forward) 5′-tgc aca ata aga gcg aac ca-3′ and (reverse) 5′-tcc tta cat tcg cat tta cct g-3′.

### Invasion assays

Invasion assays were performed in Boyden chamber inserts with Matrigel coating (Becton, Dickinson and Company, USA). Insert membranes were stained with 4′, 6-diamidino-2-phenylindole (DAPI) or a 0.1% crystal violet solution (Sigma, USA). Cells were photographed with a monochromatic Olympus camera. Analysis of intensity of transwell membrane calculated in K counts mm^2^ with Odyssey LI-COR Scanner System (LI-COR Biosciences, USA) was utilized as a measure of invasion.

### RNAi-mediated knockdown and immunofluorescence

For transient siRNA transfection, cells at 30% confluence were transfected using a control siRNA and two different Silencer select siRNAs targeting *SNAI1* (s13186 and s13187), *SNAI2* (s13128 and s13127) *PXDN* (s15388 and s15390), *NTN4* (s33971 and s33970) and *GLIS3* (s46765 and s46766) at 10 nM final concentration (Ambion, USA) with Lipofectamine RNAiMAX according to the manufacturer's protocol (Invitrogen, USA). Cells were incubated with siRNA complex for 48 hours and then fixed with 4% paraformaldehyde, stained with anti-PXDN antibody (NBP1-84316, Novus Biologicals, USA), anti-NTN4 antibody (NBP2-13680, Novus Biologicals, USA) and anti-GLIS3 antibody (H- 62, sc-135267, Santa Cruz, USA) was applied at 2.5 μg/mL, 1 μg/mL and 4 μg/mL concentrations, respectively. After primary antibodies addition, cells were incubated overnight at 4°C. Alexa flour 488 conjugated secondary antibody was added for 45 minutes at room temperature (Molecular probes, USA). Cells were counter stained with DAPI for 10 minutes and images were taken with an Olympus camera at 10× magnification.

### Immunohistochemistry and pathological evaluation

Paraffin embedded tissue slides were deparaffinised and rehydrated, endogenous peroxidise activity was blocked with 3% Hydrogen peroxide, antigen retrieval was performed in 10 mmol/L citrate buffer, and nonspecific binding was blocked with blocking reagent. Anti-PXDN antibody (NBP1-84316, Novus Biologicals, USA), anti-NTN4 antibody (NBP2-13680, Novus Biologicals, USA) and anti-GLIS3 antibody (NBP2-16668, Novus Biologicals, USA) was applied at 5 μg/mL, 1 μg/mL and 8 μg/mL concentrations, respectively and incubated overnight at 4°C, followed by 60 minute incubation with secondary anti-mouse antibody HRP (Dako). The chromogen used was 3-amino-9-ethylcarbazole (AEC). Human placenta was used as the positive control for PXDN and NTN4. Human prostate tissue was used as positive control for GLIS3. Negative control for which the primary antibody was substituted with the same concentration of mouse IgG was also used for all antibodies tested. Slides were scanned using a ScanScope XT (Aperio) and immunohistochemical reactivity was evaluated by two independent investigators.

### *In vivo* chick embryo model

Melanoma cells were treated with *PXDN, NTN4* or *GLIS3*-specific siRNAs or scrambled control siRNA as described above and labelled with CM-DiO or Dil as per manufacturer's instructions (Invitrogen, USA). Cells were grown overnight in a hanging-drop fashion to allow the formation of aggregates. Fertile chicken eggs were incubated at 38°C for 48 hours prior to transplantation. Cell aggregates consisting of similar sized small aggregates from approximately 500–800 cells were harvested and carefully injected with a glass pipette into the trunk neural tube lumen of developing chicken embryos. The eggs were then sealed with adhesive tape and re-incubated for 2 days. After incubation, embryos were removed from the eggs and fixed with 4% paraformaldehyde and whole mounts were analyzed for the localization of melanoma cells using Lumar V12 Zeiss microscope as described previously [20]. Segments of embryos containing the melanoma cells were then washed in PBS, incubated overnight in 30% sucrose at 4°C, frozen and 20μm transverse sections cut using a cryostat. Sections were rinsed in PBS, mounted in fluorescent mounting media and imaged using a Zeiss Axio M1 microscope and AxioVision software.

### Statistical analysis

Statistical comparisons of data were performed using Student's *t*-test or ANOVA in Prism software version 5.00 (GraphPad Software Inc). Comparisons of pooled mesenchymal cell lines versus epithelial cell lines and gene expression differences between control siRNA treated and knockdown treated cells were analysed using Student's two-tailed *t*-test.

Comparisons between the invasiveness of siRNA treated cells *in vitro* and *in vivo* were tested using ANOVAs, with Tukey's post-hoc test. For grouping of data, some ANOVAs were run with data pooled from multiple graphs, this is indicated in the relevant figure legends. The significance threshold was set at 0.05.

## SUPPLEMENTARY FIGURES


