# Supplementary material for: Identifying and targeting determinants of melanoma cellular invasion
Source: Oncotarget. 2016 May 9;7(27):41186–202. doi: 10.18632/oncotarget.9227 (PMC5173051; doi:10.18632/oncotarget.9227)
Supplement: Supplementary file 1 [file oncotarget-07-41186-s001.pdf]

# Identifying and targeting determinants of melanoma cellular invasion

## Supplementary Materials

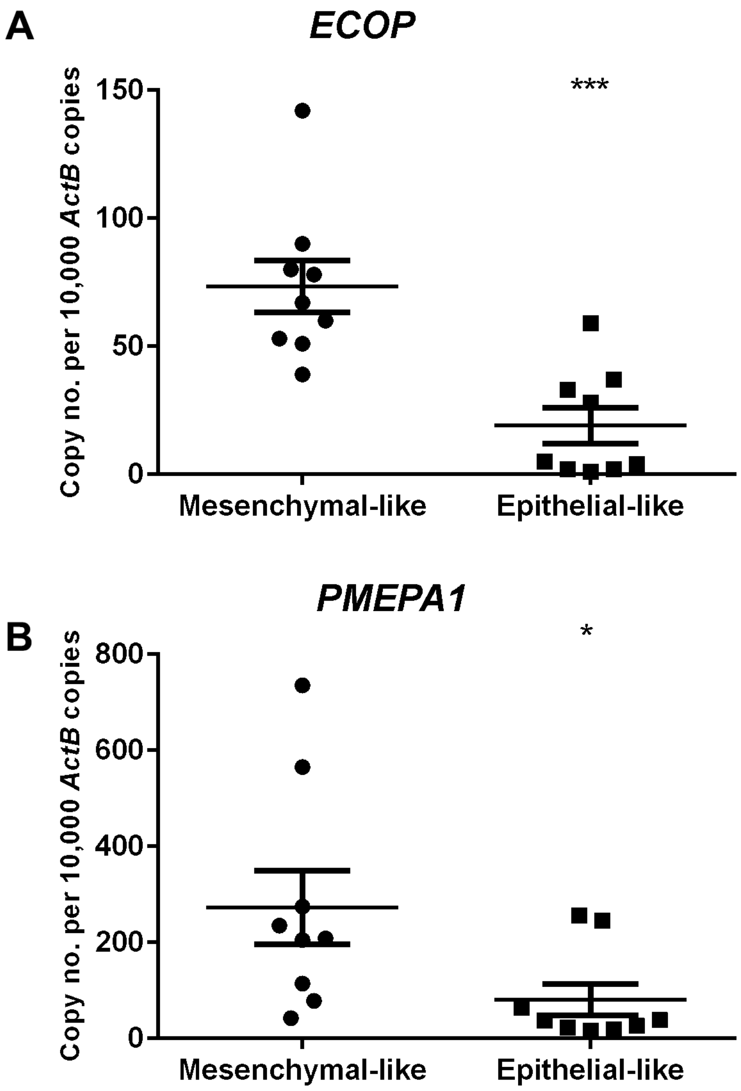


**Supplementary Figure S1: *ECOP* and *PMEPA1* expression in melanoma cell lines.** qRT-PCR analysis of (**A**) *ECOP* and (**B**) *PMEPA1* expression in a panel of metastatic mesenchymal- and epithelial-like melanoma cell lines compared to *ActB*. Bars indicate mean +/–SEM (*t*-test *$p$ = .04, ***$p$ = .0006).

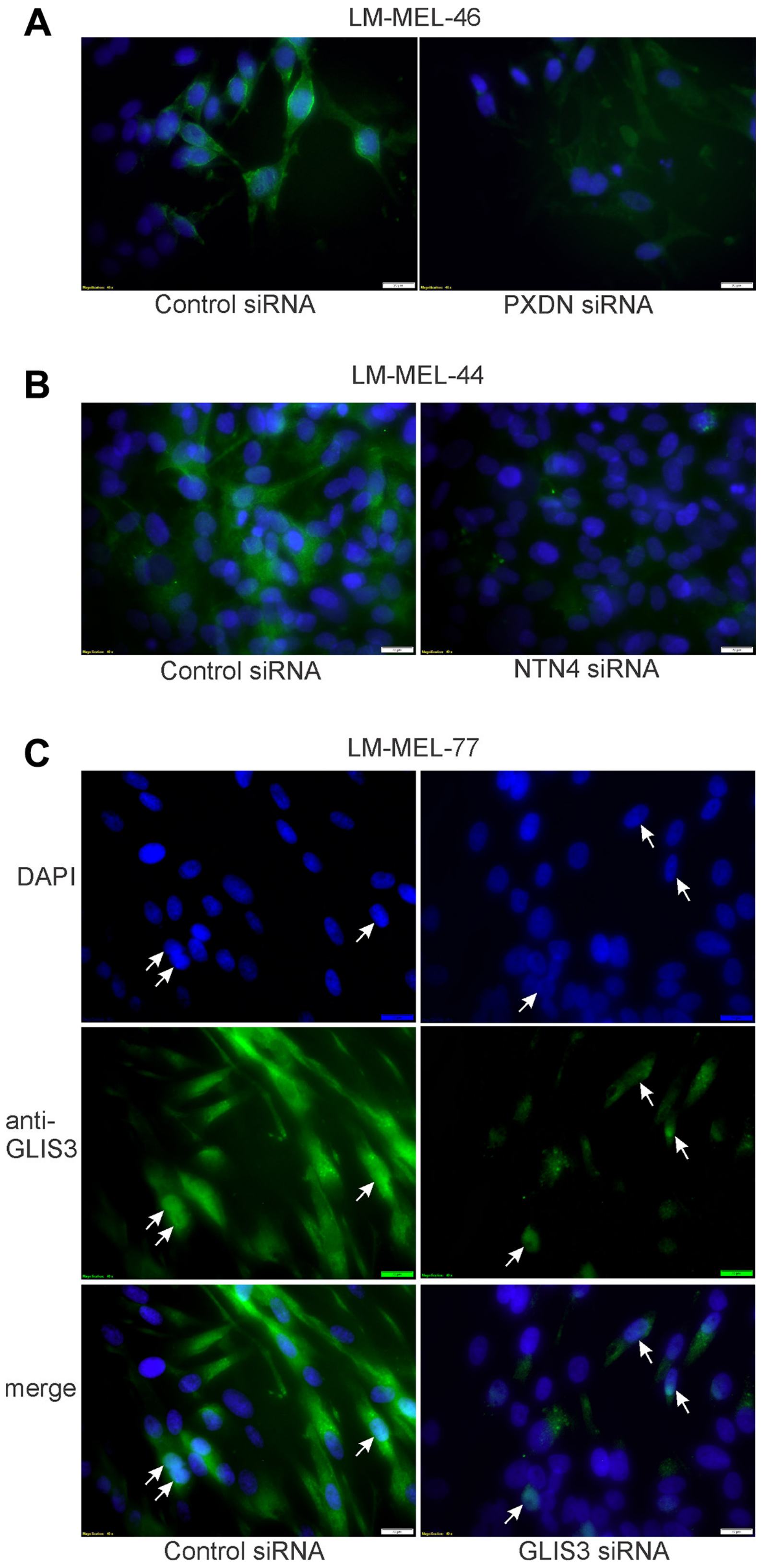


**Supplementary Figure S2: PXDN, NTN4 and GLIS3 localization in melanoma cell lines.** (**A**) After 72 h LM-MEL-46 cell line transfected with control or *PXDN* siRNA was fixed and stained using PXDN antibody in conjunction with an Alexa-488 conjugated secondary antibody and visualized with a fluorescent microscope (scale bar = 20 µm). (**B**) After 72 h LM-MEL-44 cell line treated with siRNA targeting control or *NTN4* were fixed and stained using NTN4 antibody in conjunction with an Alexa-488 conjugated secondary antibody and visualized with a fluorescent microscope (scale bar = 20 µm). (**C**) LM-MEL-77 cell line transfected with control or *GLIS3* siRNAs were fixed after 72 h and stained using GLIS3 antibody in conjunction with an Alexa-488 conjugated secondary antibody and visualized with a fluorescent microscope (scale bar = 20 µm). Arrows indicate cells with detectable nuclear GLIS3.

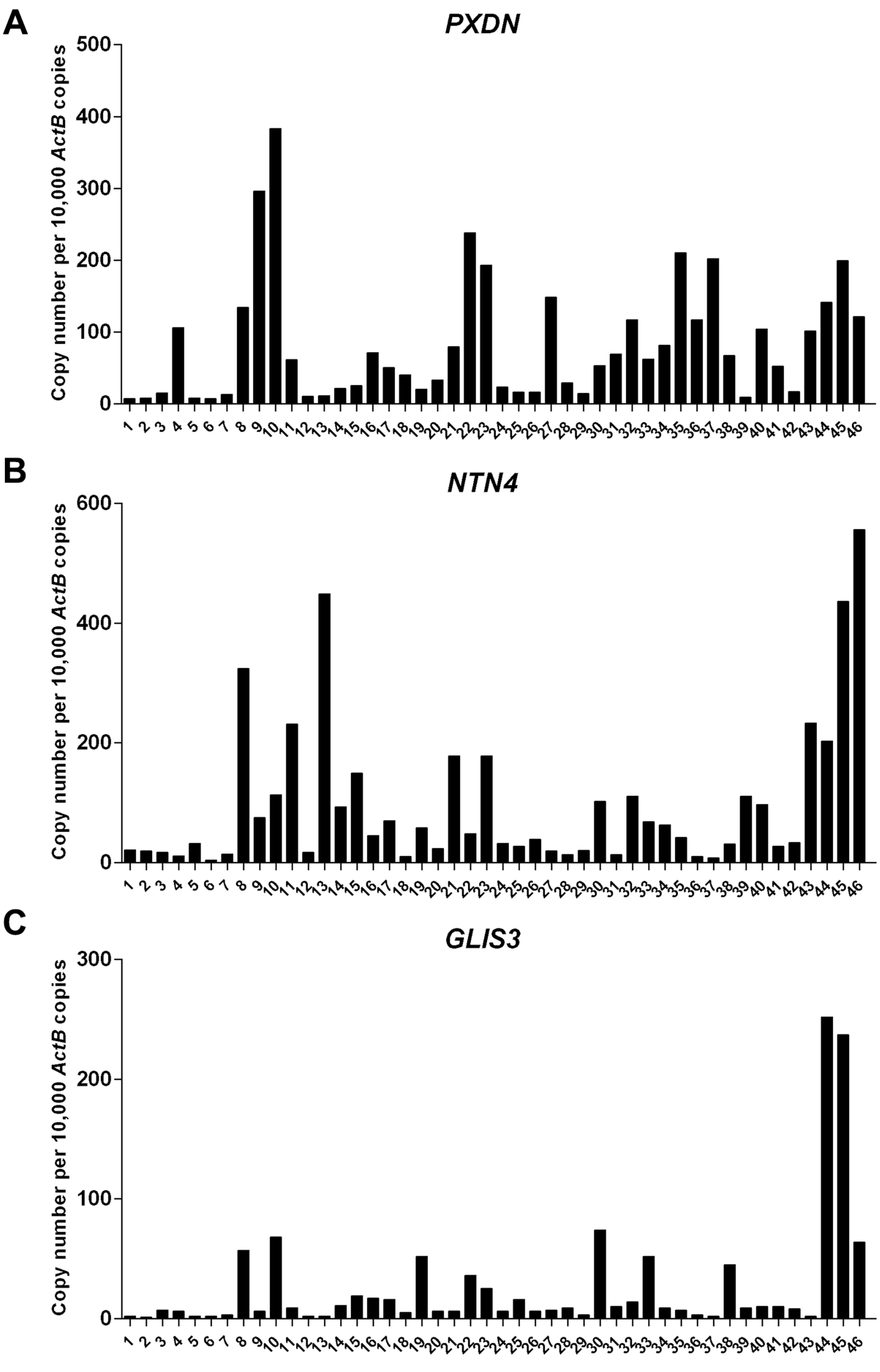


**Supplementary Figure S3: *PXDN*, *NTN4* and *GLIS3* expression in melanoma tumors.** qRT-PCR analysis of (**A**) *PXDN*, (**B**) *NTN4* and (**C**) *GLIS3* expression in a panel of metastatic melanoma tumors compared to *ActB.* The values on the x axis of each graph refer to the patient samples and expression of each gene from the same sample can be compared across the three graphs.

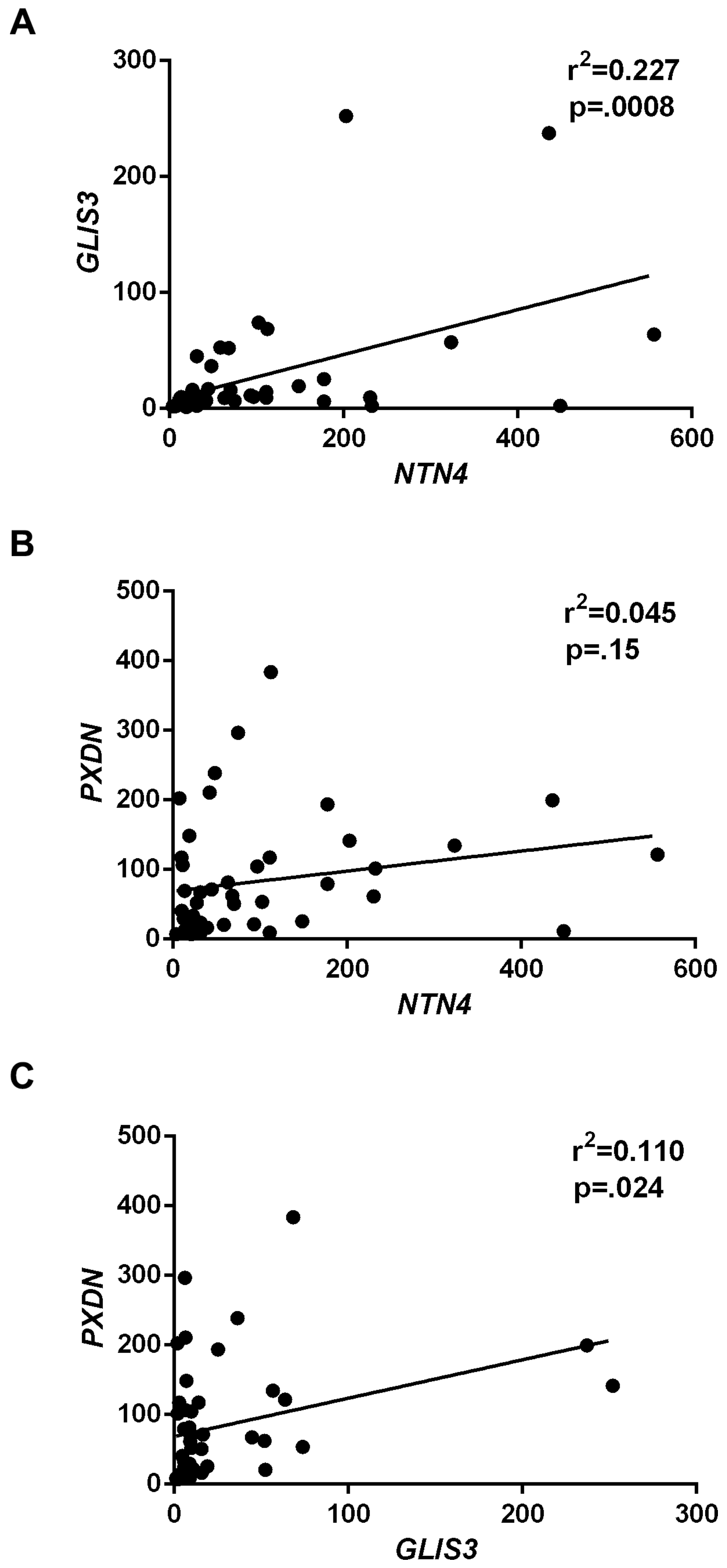


**Supplementary Figure S4: Correlation of *PXDN, NTN*4 and *GLIS3* expression in melanoma tumors.** qRT-PCR data from Supplementary Figure S3 was analysed by correlating expression of the genes for each patient sample. Regression analysis was performed for each pair. Values are copy number per 10,000 *ActB* copies. (**A**) GLIS3 and NTN4, $r^2 = 0.227$, $p = .0008$ (**B**) PXDN and NTN4, $r^2 = 0.045$, $p = .15$ (**C**) PXDN and GLIS3, $r^2 = 0.110$, $p = .\ 0244$.

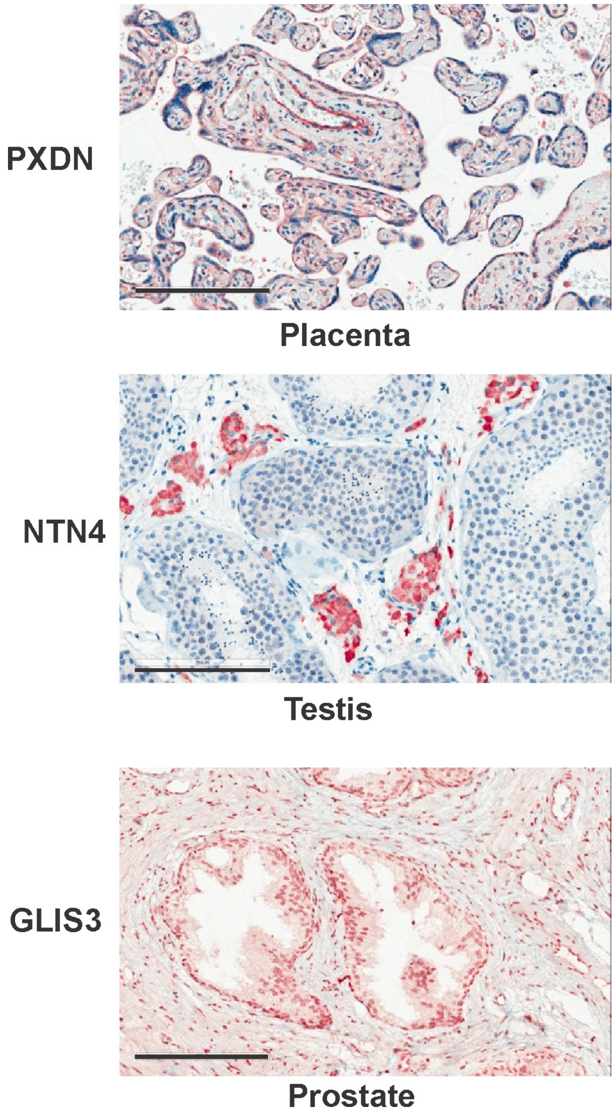


**Supplementary Figure S5: Positive control tissue localisation of PXDN, NTN4 and GLIS3.** Human placenta was used as positive control for (**A**) PXDN, testis was the positive control for (**B**) NTN4 antibodies and prostate tissue was used as positive control for (**C**) GLIS3 antibody (scale bar = 200 μm).

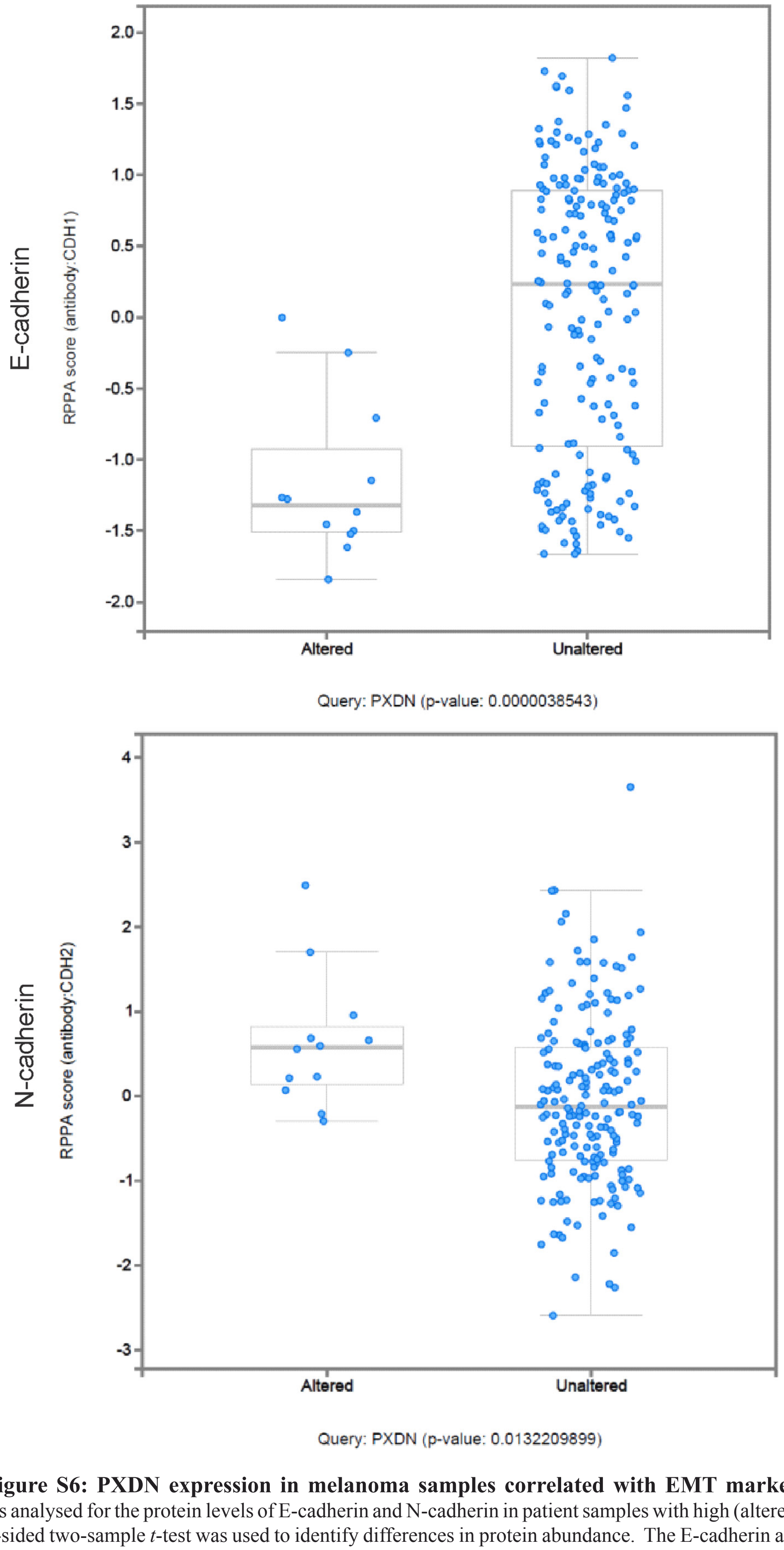


**Supplementary Figure S6: PXDN expression in melanoma samples correlated with EMT markers.** TCGA obtained melanoma dataset was analysed for the protein levels of E-cadherin and N-cadherin in patient samples with high (altered) PXDN or unaltered PXDN levels. A two-sided two-sample *t*-test was used to identify differences in protein abundance. The E-cadherin and N-cadherin protein abundance in the samples with altered and unaltered PXDN levels are displayed as a boxplot. A subset of patients with high PXDN showed significantly low E-cadherin ($p < 0.0001$) and high N-cadherin ($p = 0.013$) compared to patients with unaltered PXDN expression.

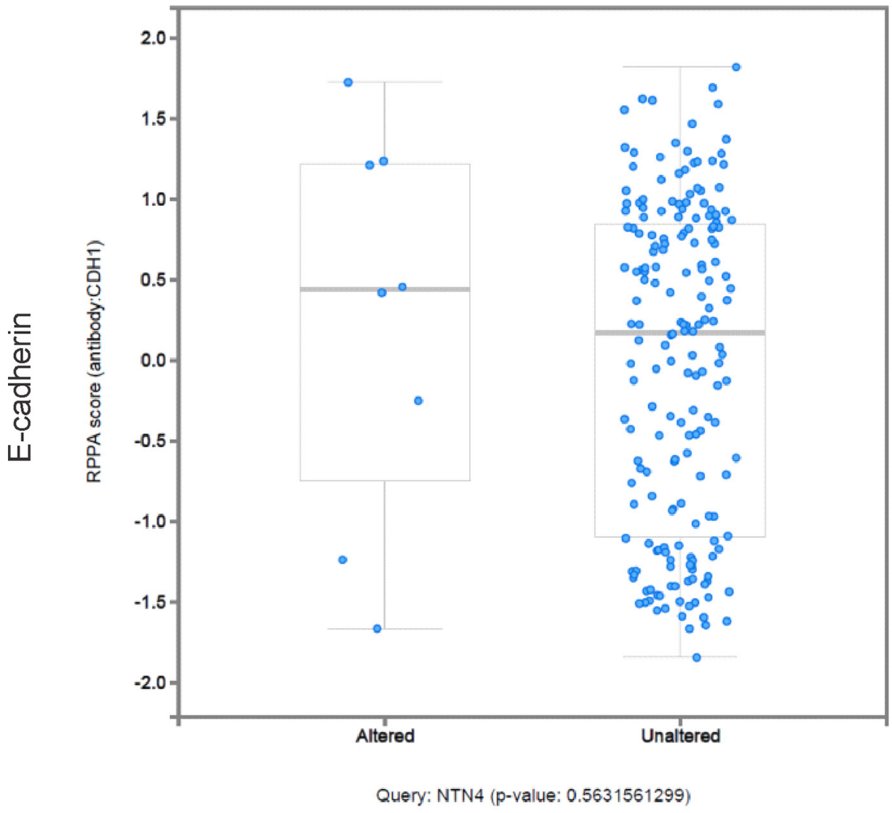


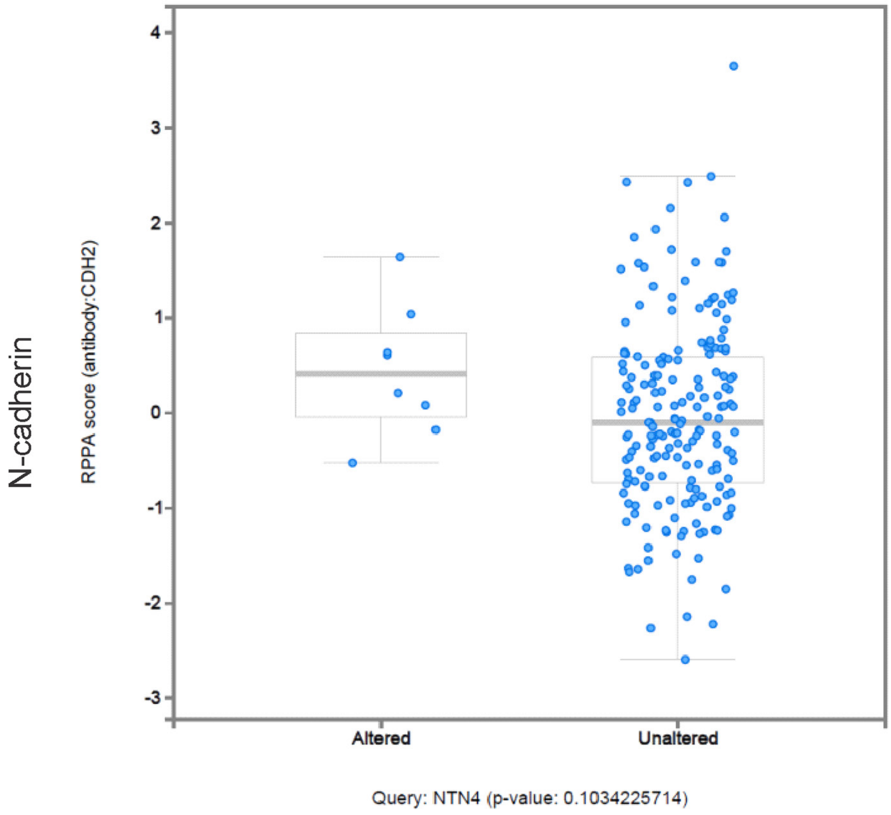


**Supplementary Figure S7: NTN4 expression in melanoma samples correlated with EMT markers.** TCGA obtained dataset was analysed for the protein levels of E-cadherin and N-cadherin in melanoma patient samples with altered (high) NTN4 levels compared to unaltered using a two-sided two-sample *t*-test. The E-cadherin and N-cadherin protein abundance in the samples with altered and unaltered NTN4 levels are displayed as a boxplot. Patients with high NTN4 showed no difference in E-cadherin ($p = 0.5$) or N-cadherin ($p = 0.1$) protein expression compared to patients with unaltered NTN4 expression.

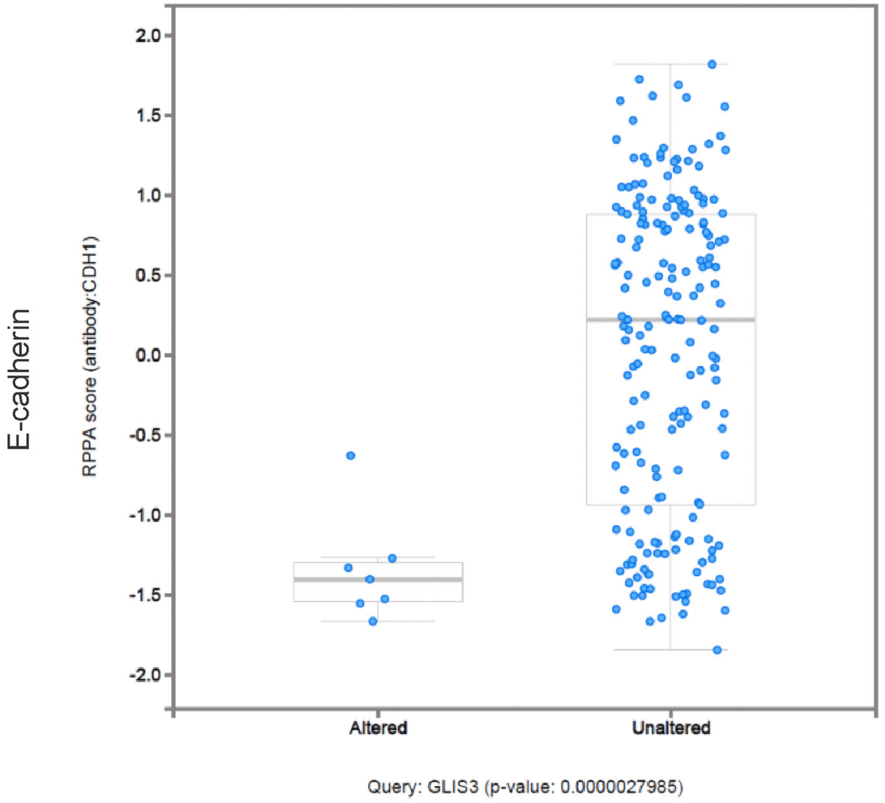


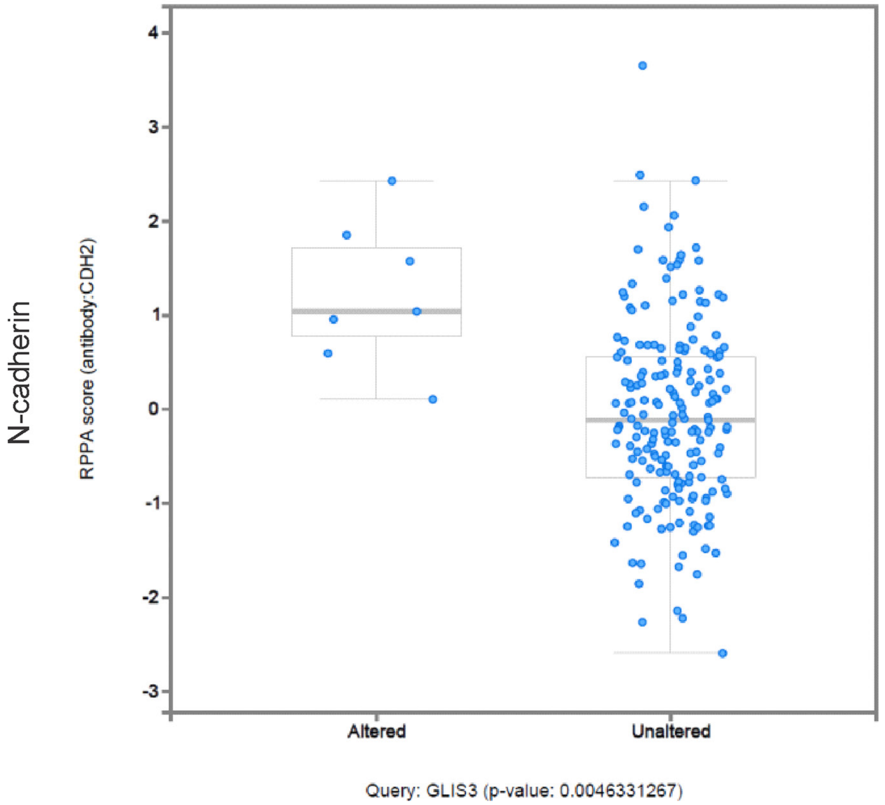


**Supplementary Figure S8: GLIS3 expression in melanoma samples correlated with EMT markers.** TCGA obtained dataset was analysed for E-cadherin and N-cadherin protein levels in melanoma patient samples with high levels of GLIS3 (altered) and unaltered samples, using a two-sided two-sample *t*-test. The E-cadherin and N-cadherin protein abundance in the samples with altered and unaltered GLIS3 levels are displayed as a boxplot. Samples with high GLIS3 showed significantly low levels of E-cadherin ($p < 0.0001$) and high N-cadherin ($p = 0.0046$) protein expression compared to samples with unaltered GLIS3 expression.
